# *Atp8* is in the ground pattern of flatworm mitochondrial genomes

**DOI:** 10.1186/s12864-017-3807-2

**Published:** 2017-05-26

**Authors:** Bernhard Egger, Lutz Bachmann, Bastian Fromm

**Affiliations:** 1 Institute of Zoology, University of Innsbruck, Technikerstrasse 25, 6020 Innsbruck, Austria; 2 Natural History Museum, University of Oslo, PO Box 1172, Blindern, 0318 Oslo Norway; 3 Department of Tumor Biology, Institute for Cancer Research, The Norwegian Radium Hospital, Oslo University Hospital, PO Box 4950, Nydalen, N-0424 Oslo Norway

**Keywords:** *atp8*, Mitochondrial genomes, Flatworms, Evolution, Gene order, Duplications, Tandem repeats

## Abstract

**Background:**

To date, mitochondrial genomes of more than one hundred flatworms (Platyhelminthes) have been sequenced. They show a high degree of similarity and a strong taxonomic bias towards parasitic lineages. The mitochondrial gene *atp8* has not been confidently annotated in any flatworm sequenced to date. However, sampling of free-living flatworm lineages is incomplete. We addressed this by sequencing the mitochondrial genomes of the two small-bodied (about 1 mm in length) free-living flatworms *Stenostomum sthenum* and *Macrostomum lignano* as the first representatives of the earliest branching flatworm taxa Catenulida and Macrostomorpha respectively.

**Results:**

We have used high-throughput DNA and RNA sequence data and PCR to establish the mitochondrial genome sequences and gene orders of *S. sthenum* and *M. lignano*. The mitochondrial genome of *S. sthenum* is 16,944 bp long and includes a 1,884 bp long inverted repeat region containing the complete sequences of *nad3*, *rrnS,* and nine tRNA genes. The model flatworm *M. lignano* has the smallest known mitochondrial genome among free-living flatworms, with a length of 14,193 bp. The mitochondrial genome of *M. lignano* lacks duplicated genes, however, tandem repeats were detected in a non-coding region.

Mitochondrial gene order is poorly conserved in flatworms, only a single pair of adjacent ribosomal or protein-coding genes – *nad4l-nad4* – was found in *S. sthenum* and *M. lignano* that also occurs in other published flatworm mitochondrial genomes. Unexpectedly, we unambiguously identified the full metazoan mitochondrial protein-coding gene complement including *atp8* in *S. sthenum* and *M. lignano*. A subsequent search detected *atp8* in all mitochondrial genomes of polyclad flatworms published to date, although the gene wasn’t previously annotated in these species.

**Conclusions:**

Manual, but not automated genome annotation revealed the presence of *atp8* in basally branching free-living flatworms, signifying both the importance of manual data curation and of diverse taxon sampling. We conclude that the loss of *atp8* within flatworms is restricted to the parasitic taxon Neodermata.

**Electronic supplementary material:**

The online version of this article (doi:10.1186/s12864-017-3807-2) contains supplementary material, which is available to authorized users.

## Background

**Fig. 1:**
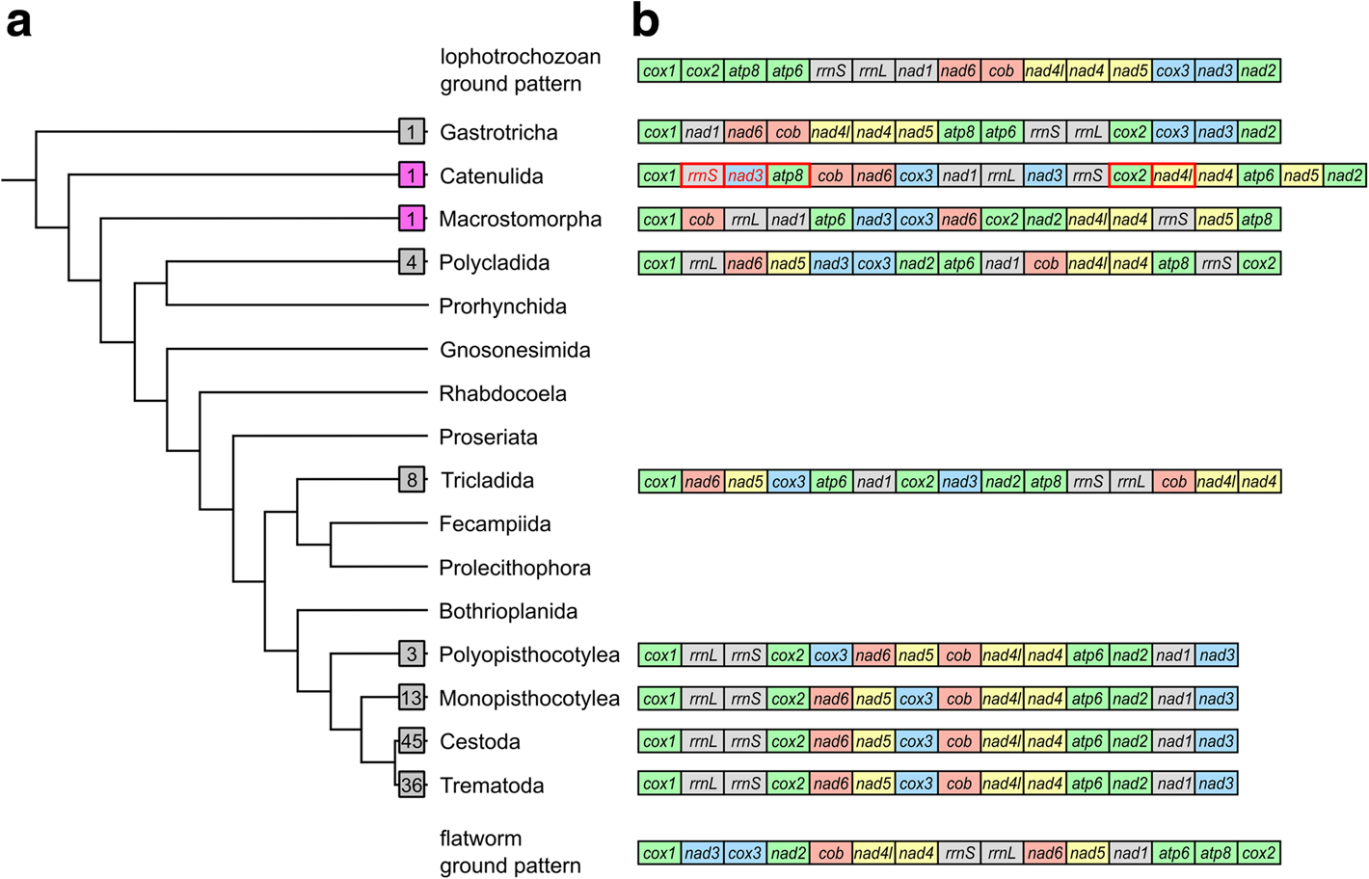
(**a**) Phylogenetic tree of Platyhelminthes with Gastrotricha as sister group reconstructed from several sources [1, 6, 7].* Squares* with *grey* background indicate the number of species with published mitochondrial genomes in the respective taxon. *Pink* background indicates newly sequenced species in this study. (**b**) Mitochondrial gene orders representative for the taxa displayed on the phylogenetic tree on the left. Only ribosomal and protein-coding genes are considered. Colours follow the widely conserved gene cartridges indicated by Mwinyi et al. [18]. Putative lophotrochozoan ground pattern after Bernt et al. [14]. Possible gastrotrich ground pattern with the single sequenced species, *Lepidodermella squamata* [1]. Possible catenulid ground pattern with the single newly sequenced species, *Stenostomum sthenum*. Duplicated genes in *red*, transcription from the minus strand is indicated by *rectangle* outlined in *red*. Possible macrostomorphan ground pattern with the single complete newly sequenced species, *Macrostomum lignano*. Possible polyclad ground pattern represented by *Prosthiostomum siphunculus* [2]. Triclad ground pattern after several sources [3–5]. Neodermatan ground patterns after Wey-Fabrizius et al. [9]. Putative flatworm ground pattern calculated with TreeREx (present study)

Flatworms represent a group with more than 100 complete mitochondrial genomes sequenced at present and thus it could be assumed that the group is well sampled. However, previous sampling strategies have focussed on the parasitic Neodermata and have not been comprehensive in free-living (turbellarian) flatworms. In particular, 97 mitochondrial genomes are available from the Neodermata, and only 12 from free-living flatworms: four species of Polycladida [1, 2] and 8 species of Tricladida [3–5], covering 2 of 11 recognised turbellarian orders (Fig. 1a). Recent phylogenomic studies have shown that the Catenulida and Macrostomorpha are the earliest branching taxa of Platyhelminthes [6, 7], but only a relatively short mitochondrial genome fragment (6,881 bp) from *Microstomum lineare* (Macrostomorpha) has been characterised [8] from either of these two groups.

Until recently, the gene order of Platyhelminthes was considered to be highly conserved, with few exceptions [9]. This notion was largely influenced by taxonomically biased sampling, as only a single complete mitochondrial genome of a free-living flatworm, *Dugesia japonica* [3], was available. When the first mitochondrial genomes of polyclads became available, a third gene order pattern within flatworms was identified [1, 2].

All previously published free-living flatworm mitogenomes represent large species, which imposes a further sampling bias. Here, we provide the first mitochondrial genomes from microscopic turbellarians representing the orders Catenulida and Macrostomorpha to shed light on the ground plan of mitochondrial genomes in Platyhelminthes. *Stenostomum sthenum* is a freshwater species from a genus with a worldwide distribution, and which predominantly reproduces asexually by fission [10]. The marine species *Macrostomum lignano* is considered a model organism whose genome has recently been sequenced [11].

## Results

### Assemblies and tandem repeats

Bioinformatic assembly delivered mitochondrial genomes with an average coverage of 1,523× (268,745 mapped DNA reads; ca. 0.9% of the original reads) in *Stenostomum sthenum*, and 440× (63,938 mapped DNA reads; ca. 0.1% of the original reads) in *Macrostomum lignano*.

**Fig. 2:**
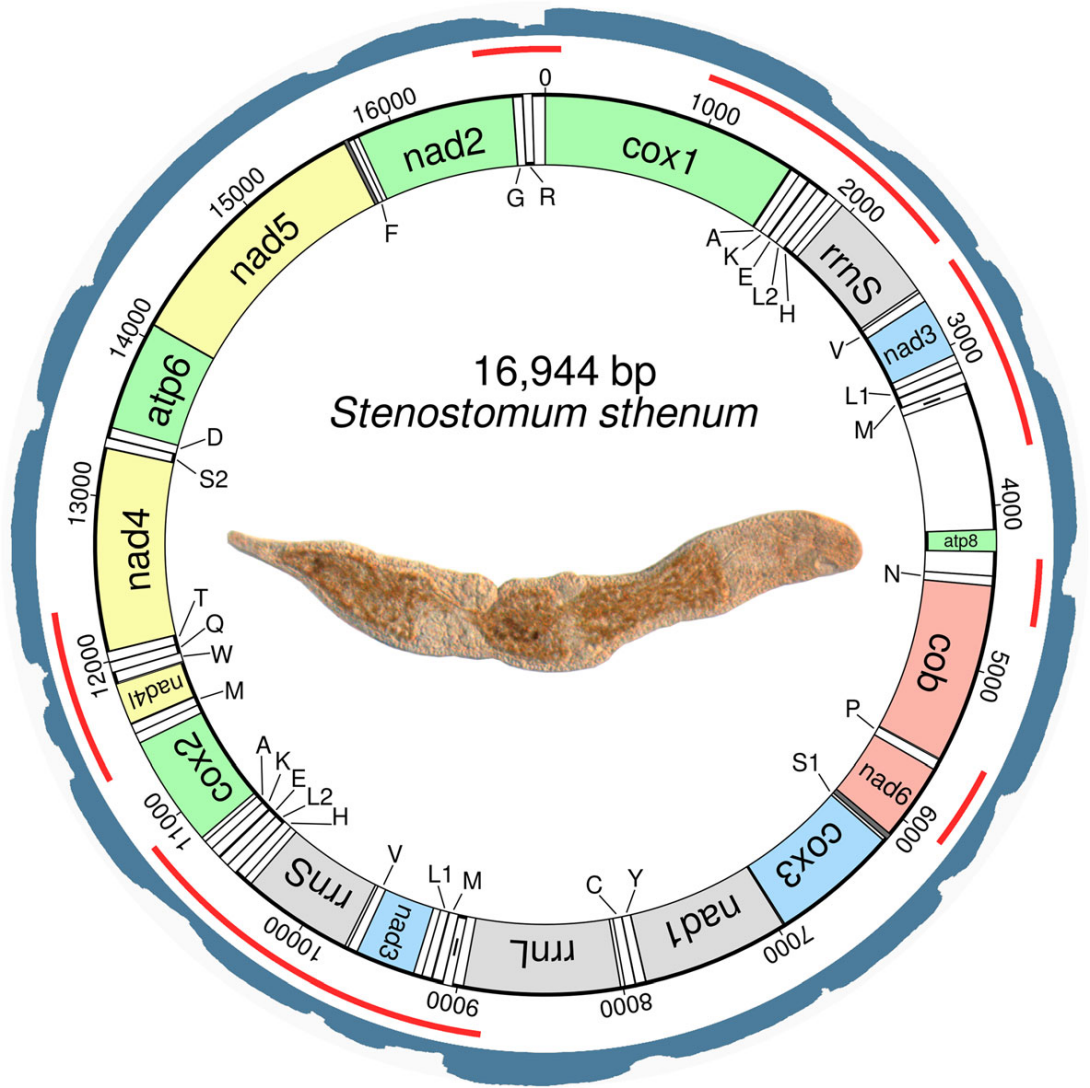
Circular mitochondrial genome of *Stenostomum sthenum*. Outermost circle displays logarithmically mapped RNAseq reads. *Red* lines below represent sequenced amplicons from PCR experiments. Bold line in innermost circle indicates transcription from the minus strand. tRNA genes are abbreviated with single capital letter and a number if applicable. Colours indicate the widely conserved gene cartridges proposed by Mwinyi et al. [18]. *Dark grey* colour indicates an overlap between genes. Unlabeled and uncoloured regions are intergenic spacers. Pictured is an adult specimen of *S. sthenum* with three zooids, anterior is to the right

The mitochondrial genome assembly of *S. sthenum* revealed an inverted region of 1,884 bp length, which is otherwise identical to its non-inverted counterpart (Fig. 2). The bioinformatically predicted flanking regions of the duplicated region were confirmed by sequencing several different PCR products. No tandem repeats were detected in the mitochondrial genome of *S. sthenum*.

**Fig. 3:**
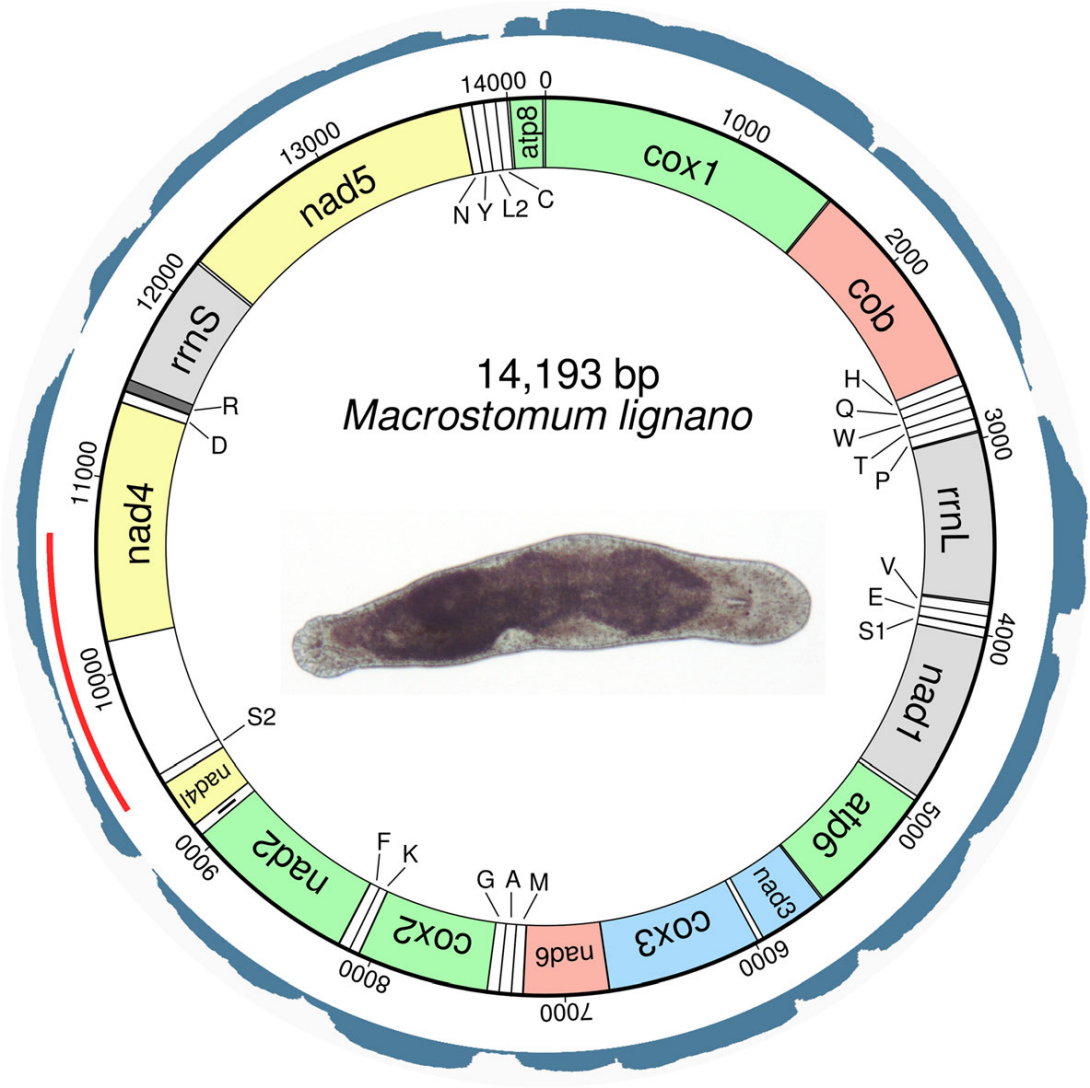
Circular mitochondrial genome of *Macrostomum lignano*. Outermost circle displays logarithmically mapped RNAseq reads. *Red* line below represents sequenced amplicons from PCR experiments. tRNA genes are abbreviated with single capital letter and a number if applicable. Colours indicate the widely conserved gene cartridges proposed by Mwinyi et al. [18]. *Dark grey* colour indicates an overlap between genes. Unlabeled and uncoloured regions are intergenic spacers. Pictured is an adult specimen of *M. lignano*, anterior is to the right

The assembled mitogenome of *M. lignano* (Fig. 3) was compared to the published genome assembly [11], and a matching contig of 15,041 bp length (Additional file 1) with non-overlapping ends was detected. This contig allowed us to determine the length and sequence of a tandem repeat region consisting of eight 61 bp repeats. The sequence of the repeat units is GGGGGGAATAAATATTCCCCCCTATATTACTAATGTAATATAAGGAGAAATATAATATATA (Additional file 1). The 15,041 bp long genome contig showed a leading (G)_5_ sequence in repeats #4, 5 and 8, but PCR verification suggested that all 8 repeats have a leading (G)_6_ sequence.

Tandemly repeated elements are common in the mitochondrial genomes of flatworms, and a re-analysis of the published mitochondrial genome of *Prosthiostomum siphunculus* [2] revealed several large, previously undetected overlapping tandem repeats (positions 14,695-15,122) in an intergenic region.

### RNAseq read mapping on assembled mitochondrial genomes

We logarithmically mapped 21.47 million RNAseq reads onto the mitochondrial genome of *S. sthenum* with an average coverage of 126,712× (Fig. 2). For *M. lignano*, 0.80 million RNAseq reads mapped onto the mitochondrial genome with an average coverage of 5,637× (Fig. 3), and for *P. siphunculus* 0.66 million RNAseq reads resulted in an average coverage of 4,348× (Additional file 2). In all species, the highest expression levels (by a factor of about 4-50 as compared to protein coding genes) were found for *rrnL*, and all genes were found to be transcriptionally active. In *S. sthenum*, even the large non-coding regions between positions 3,307 and 4,140, and between positions 4,277 and 4,424 (Additional file 3) appear to be transcribed at high levels (Fig. 2), while in *M. lignano* the non-coding region between positions 9,444 and 10,165 (Additional file 3) is only weakly expressed (Fig. 3). In *P. siphunculus*, a large non-coding region between *rrnL* and *nad6* [2] is quite strongly expressed (Additional file 2). Generally, tRNAs show the lowest level of mapped RNAseq reads (Figs. 2 and 3, Additional file 2).

### Non-coding regions and tRNAs

The largest non-coding region in *S. sthenum* is located between *tRNA I* and *atp8*, and has a length of 840 bp (Fig. 2, Additional file 3). This region includes a small (110 bp) part of the duplicated piece containing *nad3* and *rrnS*.

We found the full complement of 22 tRNAs in *S. sthenum*, in addition to 8 duplicated (namely *tRNAs A, E, H, I, K, L1, L2* and *V*) and one triplicated (*tRNA M*) (Fig. 2,Additional file 3). In contrast, the mitochondrial genome of *M. lignano* encodes only 21 tRNAs, as a homologue of *tRNA L1* was not detected by either MITOS or ARWEN (Figs. 3, Additional file 3). Secondary structures of all tRNAs predicted by MITOS are shown in Additional files 4 and 5.

### Protein-coding genes

**Fig. 4:**
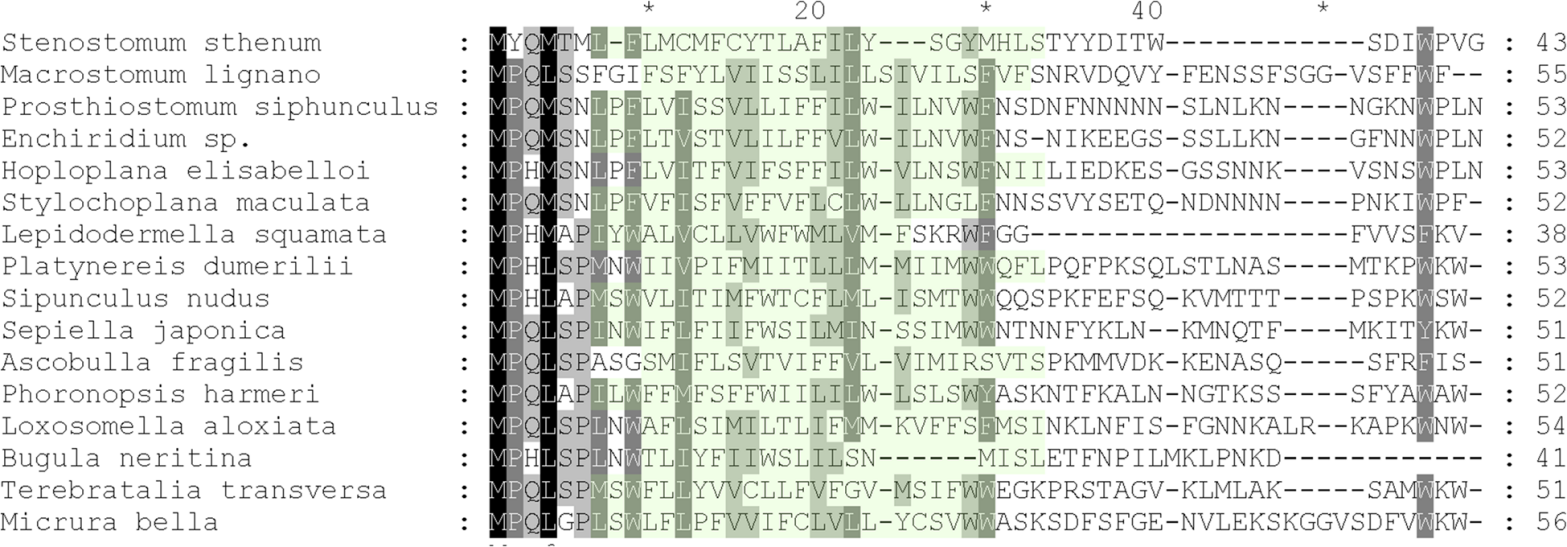
Amino acid alignment of atp8 in flatworms and other lophotrochozoans: a gastrotrich (*Lepidodermella squamata*), annelids (*Platynereis dumerilii* and *Sipunculus nudus*), molluscs (*Sepiella japonica* and *Ascobulla fragilis*), a phoronid (*Phoronopsis harmeri*), a kamptozoan (*Loxosomella aloxiata*), a bryozoan (*Bugula neritina*), a brachiopod (*Terebratalia transversa*) and a nemertean (*Micrura bella*). Transmembrane regions are underlaid in *light green*. Putative triclad atp8 sequences were omitted because of their derivedness (for inclusion of triclad sequences, see Additional file 9)

We detected all 13 protein-coding genes in the mitochondrial genomes of *S. sthenum* and *M. lignano*. This set included *atp8* both in newly sequenced species and also in the four published polyclads [1, 2]. In all but one polyclad, the starting amino acid sequence is MPQM, while in *Hoploplana elisabelloi* it is MPHM. In *S. sthenum*, the atp8 amino acid sequence starts with MYQM, in *M. lignano* with the widely conserved MPQL (Fig. 4). We performed BLASTP searches with all 6 identified flatworm atp8 amino acid sequences against NCBI's non-redundant protein sequences database, and recovered e-values between 0.027 and 0.22 for atp8 hits (except for the atp8 sequence of *S. sthenum*, which did not return any e-values below 1). To provide context for these e-values, we used BLASTP with the published derived atp8 sequences of the gastropod *Ascobulla fragilis* and the gnathostomulid *Gnathostomula armata*, where the lowest e-values compared to other atp8 sequences are 0.22 and 0.23, respectively. Similarly, the atp8 sequence of *Mytilus edulis* is only similar to other *Mytilus* species, but not to any other atp8 sequences.

Using SMART [12], we detected transmembrane regions in putative and published atp8 sequences (Fig. 4). SMART also reported the detection of protein family (Pfam) ATP-synt_8 in the putative atp8 sequences of the polyclads *P. siphunculus* and *S. maculata*, with e-values of 0.0073 and 0.72, respectively. For comparison, the published atp8 sequences of the gastrotrich *Lepidodermella squamata* and of the nemertean *Micrura bella* scored Pfam ATP-synt_8 *e*-values of 0.035 and 0.022, respectively.

The hydrophobicity profiles between the putative flatworm and two published atp8 amino acid sequences provide very similar graphs, with positive hydrophobicity scores at the N-terminus and mostly negative scores at the C-terminus (Additional file 6).

We found the identified *atp8* sequences of *S. sthenum*, *M. lignano* and *P. siphunculus* also by BLASTN searches in their respective transcriptomes, providing additional evidence that these sequences are expressed.

A manual search for *atp8* in non-coding regions, as well as using BLAST with the putative *atp8* sequences of free-living flatworms in published mitochondrial genomes from Neodermata species did not detect any homologous genes in this group of flatworms.

### Gene order

The mitochondrial ribosomal and protein-coding gene orders of the two newly sequenced species and several published species are shown in Fig. 1b.

An analysis of gene order rearrangements with CREx [13] suggests that at least four steps (all inversions, called 'reversals' in CREx) are required to change from the putative lophotrochozoan ground pattern [14] to the gene order of the single published representative of Gastrotricha, *Lepidodermella squamata* [1] (Additional file 7). Similarly, it takes four steps from the putative lophotrochozoan ground pattern to *M. lignano*, and seven steps to the *S. sthenum* gene arrangement. The fewest steps from the lophotrochozoan ground pattern to any known free-living flatworm mitochondrial genome is two in the Tricladida (both tandem duplication, random loss events) (Additional file 7).

TreeREx calculates likely ancestral gene orders in nodes of a given phylogenetic tree [15], which we provided (Fig. 1a) for the taxa where mitochondrial gene order data is available. TreeREx suggests an ancestral gene order for Platyhelminthes (Fig. 1b), which is identical to the ancestral gene order of Rhabditophora (Additional file 7). According to the TreeREx analysis, the gene order of the polyclad *Prosthiostomum siphunculus* used in Fig. 1b is the same as the putative ancestral gene order of Polycladida as well as of Trepaxonemata (all flatworms except Catenulida and Macrostomorpha) (Additional file 7). TreeREx and CREx only accept gene matrices with a full complement of genes in all taxa as input, consequently we were not able to include Neodermata (which lack *atp8*) in these analyses.

## Discussion

### Mitochondrial genome sizes and nucleotide composition

Mitogenome sizes of polyclads vary between 14,651 bp (*Enchiridium* sp.) and 15,329 bp (*Stylochoplana maculata*) [1, 2], while those of triclads are generally larger and range between 14,909 bp (*Obama* sp.) and 27,133 bp (*Schmidtea mediterranea*, sexual biotype) [4, 5]. *Macrostomum lignano*, with 14,193 bp, has the smallest mitochondrial genome of a free-living flatworm studied so far. Only neodermatans are smaller, in particular the mitochondrial genomes of the cestodes rarely exceed 14,000 bp [9]. A survey of published mitochondrial genomes shows that the sizes of cestode mitogenomes are within a narrow range between 13,387 bp (*Taenia pisiformis*) and 13,900 bp (*Hymenolepis diminuta*), with only two exceptions larger than 14,000 bp, (*Pseudanoplocephala crawfordi* 14,192 bp and *Anoplocephala perfoliata* 14,459 bp). Trematode mitogenomes are on average slightly larger, ranging between 13,796 bp (*Clinostomum complanatum*) and 15,258 bp (*Metagonimus yokogawai*) in size, with one notable exception of 16,901 bp (*Schistosoma spindale*). In polyopisthocotyleans and monopisthocotyleans, mitogenome sizes are between 13,392 bp (*Tetrancistrum nebulosi*) and 15,527 bp (*Polyabris halichoeres*).

### Gene order

The mitochondrial gene order differs between the two basally branching flatworms in this study, lacking any shared pairs of protein-coding or ribosomal gene boundaries other than *nad4l-nad4* (although *nad4l* is transcribed from the minus strand in *S. sthenum*) and a transposed *rrnL-nad1* gene pair (Fig. 1b).

While in gastrotrichs the putative lophotrochozoan ancestral gene order is largely recognisable (Fig. 1b), the extensive gene duplication in *Stenostomum* makes comparisons difficult. In fact, the only remaining adjacent gene pairs between the gastrotrich *Lepidodermella squamata* and *S. sthenum* are *nad4l-nad4* and *cob-nad6*. The gene pair *nad4l-nad4* is shared throughout the flatworms (Fig. 1b).

Within Macrostomorpha, the only full-length mitochondrial genome available is that of *M. lignano* (Fig. 3). The partial mitochondrial genome of *Microstomum lineare* is notably different from *M. lignano*, however (Additional file 8), hinting at non-conserved mitochondrial gene orders within Macrostomorpha, similar to the situation found in Polycladida, where *Stylochoplana maculata* has a completely different gene order from that of other polyclad representatives [1, 2].

Between *M. lignano* and two of the four published polyclads (the cotyleans *Prosthiostomum siphunculus* and *Enchiridium* sp.), the only identical adjacent gene pairs are *nad3-cox3*, *nad4l*-*nad4*, and *nad1-atp6*. Not surprisingly, the gene order in *M. lignano* requires the lowest number of rearrangements from the inferred ancestral gene order in flatworms, while the gene order of *P. siphunculus* may closely reflect the ancestral gene order of trepaxonematans (Additional file 7).

We have noticed some confusion about gene content and order in the partial mitochondrial genome of *Microstomum lineare*, originally published by Ruiz-Trillo et al. [8]. In the study of Golombek et al. [1], an additional gene, *nad6*, was shown in their Figure 7. Our own analysis suggests this may be an artifact of an inaccurate MITOS annotation, which weakly supports *nad6* in the genome fragment, but further BLAST searches show this to be a very unlikely *nad6* candidate. In the study of Aguado et al. [2], the gene order of *M. lineare* is shown inverted in their Fig. 2. In our own figure (Additional file 8), we depict the original gene order and content [8].

The gene block *nad6*, *nad5* and *cox3* was suggested to be a possible plesiomorphy for the Rhabditophora [2], but it is not found in both studied macrostomorphans or the catenulid *S. sthenum* (Fig. 1b, Additional file 8) and is thus more likely an apomorphy for Trepaxonemata, which is supported by the TreeREx analysis (Additional file 7). Another possible trepaxonematan apomorphy is the block *cob*, *nad4l* and *nad4* (Fig. 1b), also corroborated by TreeREx (Additional file 7). The gene pair *cox3* and *nad3* is ancestral in bilaterians, in lophotrochozoans and also in gastrotrichs (Fig. 1b) [1, 14] the likely sister group of Platyhelminthes [6, 16, 17]. Within flatworms, a close association between *cox3* and *nad3* - either as *cox3-nad3* or *nad3-cox3* is conserved within most polyclads and also in *Macrostomum*, and is thus likely a plesiomorphy in these groups (Fig. 1b, Additional file 7). In all other flatworms, including *S. sthenum* and *M. lineare*, *cox3* and *nad3* are not adjacent, suggesting an independently derived character state.

Ribosomal gene adjacency (*rrnS* and *rrnL*) is very common in bilaterians and lophotrochozoans [14] and appears to be conserved in *M. lineare*, in triclads and neodermatans (Fig. 1b, Additional file 8). As noted by Aguado et al. [2], the two ribosomal genes are not adjacent in polyclads, and this is also true for *S. sthenum* and *M. lignano* (Figs. 1b, 2 and 3). A parsimonious explanation for these findings is multiple, independent loss of ribosomal gene adjacency. This solution is supported by the relative vicinity of *rrnS* and *rrnL* in *S. sthenum*, only interrupted by *nad3* (Fig. 2). Alternatively, the adjacency of ribosomal genes was lost for all flatworms and was regained in *M. lineare* and in Trepaxonemata.

The location of *tRNA V* between *rrnS* and *rrnL* is an ancestral and widespread condition [18] and commonly found also in lophotrochozoans, including the gastrotrich *Lepidodermella squamata* [1]. This feature was lost in the common ancestor of the Platyhelminthes, as it is not found in any of the 109 flatworms sequenced so far, including those flatworm groups where *rrnS* and *rrnL* are still adjacent. In *S. sthenum*, both *rrnS* and *tRNA V* are duplicated and adjacent to each other (Fig. 2, Additional file 3), which is likely a plesiomorphy. In *M. lignano tRNA V* is located downstream of *rrnL* (Fig. 3, Additional file 3), probably the result of a transposition. In the other sequenced macrostomorphan, *M. lineare*, both ribosomal genes are present in the published mitochondrial genome fragment, but *tRNA V* was not identified between the ribosomal genes [8].

Our CREx and TreeREx analyses (Additional file 7) suggest that the gene order of *S. sthenum* is highly derived; possibly representatives of other Catenulida families could shed light on the ancestral mitochondrial gene order of Catenulida. Similarly, the ancestral gene order of Macrostomorpha cannot be deduced from the available data, thus more representatives of early branching flatworms should be analysed.

### *Atp8* is in the ground pattern of flatworms

*Atp8* has been noted as missing from the mitochondrial genomes of flatworms [1, 2, 4, 9, 19], but it was annotated in the single sequenced gastrotrich, *Lepidodermella squamata* [1]. Annotation of atp8 is particularly difficult, as its amino acid sequence - apart from the first four amino acids MPQL - is not well conserved, and it has been reported as lacking in nematodes, chaetognaths, rotiferans and bivalve molluscs [19]. Recently it was suggested that an extremely derived *atp8* may be present in triclad flatworms (planarians) [5]. The authors based their argument on the fact that their putative *atp8* was expressed in RNAseq reads and that the sequence codes for a protein with putative transmembrane domains, like *atp8*. However, no pronounced similarity with *atp8* sequences from other animals was found (see also Additional files 9, 10 and 11), and the putative *atp8* sequences were annotated with a single question mark instead of a specific gene name [5].

As automated gene annotation with MITOS [20] could not identify *atp8* neither in our two basally branching flatworms nor in three of the four published polyclad mitochondrial genomes (only in *P. siphunculus* does MITOS correctly annotate *atp8*, but it was not annotated by the original authors [2]), we have manually searched for *atp8* candidates in all six mitochondrial genomes. In this way, we were able to identify *atp8* in *Stenostomum*, *Macrostomum* and in all four published polyclads (Fig. 4). The presence of *atp8* in these mitochondrial genomes is supported by RNAseq read mapping (Figs. 2, 3 and 4, Additional file 2), transmembrane regions at the N-terminus (Fig. 4), hydrophobicity profiles (Additional file 6) and, weakly, by BLAST and Pfam e-values. This implies that *atp8* has not been lost in all flatworms, but only in the parasitic Neodermata, and it also supports the notion that the extremely derived *atp8* candidate found in triclads may be a functional *atp8* [5]. This case also highlights the importance of manual curation following automated annotation, and that observation bias (“no *atp8* in flatworms”) can lead to oversight.

### Multiple gene duplications and transcription from the minus strand in *Stenostomum*

With *S. sthenum*, we present the first mitochondrial genome of a flatworm with both extensive duplication (including triplication of tRNA genes, duplication of a ribosomal and a protein-coding gene) and transcription from the minus strand of a substantial number of genes (Figs. 1b, 2). So far, only tRNA genes have been reported as being duplicated in mitochondrial genomes of some flatworms, namely *tRNA N* in the triclads *Dugesia japonica* and *D. ryukyuensis* [3] and *tRNA C* in the two parasitic flatworms *Schistosoma mansoni* and *S. japonicum* [9]. The highly unusual features of *S. sthenum* most likely represent a derived condition, as neither gastrotrichs, nor the inferred lophotrochozoan ground pattern show any similarity to *Stenostomum* in this regard (Fig. 1b).

### Large non-coding RNA

RNAseq reads were mapped onto the mitochondrial genome of several triclad flatworms [5], providing evidence for large (>100 bp) regions that were transcriptionally active, but not coding. Here we have mapped RNAseq reads to large non-coding regions of *S. sthenum*, *M. lignano* and *P. siphunculus* (Figs. 2 and 3, Additional file 2), supporting the idea that large non-coding RNA is not restricted to vertebrates [5].

Tandem repeats in both *M. lignano* and *P. siphunculus* occur in their large non-coding regions and such sequences have been hypothesised to represent control regions and/or potential origins of replication [3]. Tandem repeats in non-coding regions are also very common in parasitic flatworms [21]. Interestingly, in *S. sthenum* there are no tandem repeats, suggesting that the putative control region is not uniformly structured in flatworms, and that tandem repeats were either lost in catenulids or are an apomorphy for Rhabditophora.

## Conclusions

We have sequenced two mitochondrial genomes from the earliest branching flatworm taxa, Catenulida (*Stenostomum sthenum*) and Macrostomorpha (*Macrostomum lignano*). *S. sthenum* shows a highly unusual duplication pattern including a ribosomal, a protein-coding and several tRNA genes, and has several genes transcribed from the minus strand. *M. lignano* has a markedly different gene order from that known from the partial mitochondrial genome of another macrostomorphan, *Microstomum lineare*. Even with our newly sequenced species, mitochondrial genome data are still lacking from half of flatworm orders (Fig. 1a). With the currently available information on flatworm mitochondrial genomes, only a preliminary ancestral mitochondrial gene order for flatworms can be inferred. More representatives of early divergent, non-parasitic taxa such as Catenulida and Macrostomorpha, but also from the 7 as yet unsequenced flatworm orders are required to obtain a comprehensive picture of mitochondrial genome evolution in flatworms. Another major conclusion of the present study is that *atp8* is present in several and possibly all free-living flatworms, and that *atp8* has been lost only in the parasitic Neodermata.

Manual curation of automatically annotated mitochondrial genomes is still very important. Without meticulous analysis of new and published mitochondrial genomes of free-living flatworms, and the combined use of RNA and DNA data, *atp8* may not have been found and annotated in flatworm mitochondrial genomes.

A very recent paper on the mitochondrial genome of the closely related catenulid *Stenostomum leucops* [22] has annotated a divergent *atp8* gene, which supports the findings reported here for *S. sthenum*.

## Methods

### Animals

*Stenostomum sthenum* and *Macrostomum lignano* have been kept as laboratory cultures since 2009 and 1995, respectively. For *S. sthenum*, a clonal line started from a single animal collected in Innsbruck, Austria, and for *M. lignano*, non-inbred culture animals [23] have been used for generating the RNAseq reads as described by Egger et al. [6] as well as for total genomic DNA extraction for next-generation sequencing (NGS) and PCR experiments.

### DNA extraction and Illumina sequencing

About 100 specimens of clonal *S. sthenum* and *M. lignano*, respectively, were pooled for total genomic DNA extraction using the E.Z.N.A. Tissue DNA kit (Omega Bio-Tek) following the Tissue DNA-Spin protocol. Next-generation sequencing was performed on an Illumina HiSeq 2000 instrument delivering 33,362,560 (*S. sthenum*) and 54,410,350 (*M. lignano*) 100 bp paired-end reads that were subsequently trimmed and QC filtered using the fastx-toolkit (hannonlab.cshl.edu/fastx_toolkit/), leaving 29,990,734 (*S. sthenum*) and 52,268,272 (*M. lignano*) 100 bp paired-end reads for assembly. The custom sequencing service was provided by the Norwegian Sequencing Centre (www.sequencing.uio.no).

### Assembly

The mitochondrial genome of each species was reconstructed by directly assembling the NGS reads using the mitochondrial baiting and iterative mapping algorithm MITObim 1.8 [24] with the default settings of the program, and the optional quality trimming option *trimreads* (see the program’s tutorial at https://github.com/chrishah/MITObim for details). The partial cytochrome oxidase subunit 1 sequences of *S. leucops* (GenBank accession number AJ405976) and *M. lignano* (GenBank accession number KP308282), respectively, were used as starting seeds for the assemblies.

### BLAST search in transcriptomes

The BLAST+ suite [25] with published protein-coding mitochondrial genes as queries was used to screen the transcriptomes [6] of *S. sthenum* and *M. lignano*. Furthermore, all genes annotated from the MITObim assemblies were used for BLAST searches of the respective transcriptomes.

### DNA extraction for PCR

DNA was extracted from 100-220 animals per species with phenol:chloroform:isoamylalcohol or a QIAamp DNA Micro Kit (Qiagen), eluted in nuclease-free water or buffer AE. PCRs were done with 15-170 ng of extracted DNA in 50 μl tubes, with varying conditions.

### Annotation and gene order

For gene annotation, BLAST searches [26], as well as MITOS [20], ARWEN [27] and DOGMA [28] predictions were employed. For the analysis of putative atp8 sequences, we also used SMART [12] with default parameters to identify domains, and the ExPASy tool ProtScale (http://web.expasy.org/protscale/) to determine hydrophobicity profiles of amino acid sequences. Gene order rearrangements were analysed with CREx [13] and TreeREx [15]. Tandem Repeats Finder was used to identify tandem repeats [29].

### Mapping RNAseq reads to the mitochondrial genomes

Bowtie2 [30] and samtools [31] were used with default parameters to map RNAseq reads [6] to the assembled mitochondrial genomes of *S. sthenum*, *M. lignano* and the published mitochondrial genome of the polyclad *Prosthiostomum siphunculus* [2]. Mapped reads were visualised with the Integrative Genomics Viewer [32]. Figures were made with mtviz (pacosy.informatik.uni-leipzig.de/mtviz), Inkscape (www.inkscape.org) and GIMP (www.gimp.org).

### Amino acid alignments

For multiple gene alignments of atp8, we have used MAFFT [33] with the parameters “-l -o ' --inputorder --auto ' -a 50 -e 1.0e-10 -s input” on amino acid sequences of our own sequences, downloaded sequences from NCBI of lophotrochozoan atp8 proteins and putative atp8 sequences in triclads [5]. Accession number of used sequences are: AKD00049 (*Lepidodermella squamata*), AAF02682 (*Platynereis dumerilii*), ACJ11904 (*Sipunculus nudus*), BAM11165 (*Sepiella japonica*), ACE62808 (*Ascobulla fragilis*), AES86298 (*Phoronopsis harmeri*), BAG12588 (*Loxosomella aloxiata*), AJP00039 (*Bugula neritina*), AAK95499 (*Terebratalia transversa*) and AKT74029 (*Micrura bella*). For visualisation of alignments, we have used GeneDoc (http://genedoc.software.informer.com/2.7/).

## Additional files

Additional file 1: Mitochondrial genome sequence from the assembled nuclear genome of *Macrostomum lignano* [11]. (TXT 14 kb)

Additional file 2: Circular mitochondrial genome of *Prosthiostomum siphunculus* [2]. Outermost circle displays logarithmically mapped RNAseq reads. tRNA genes are abbreviated with tRNA and three letters and a number if applicable. Colours indicate the widely conserved gene cartridges proposed by Mwinyi et al. [18]. Unlabeled and uncoloured regions are intergenic spacers. (TIF 870 kb)

Additional file 3: Gene annotation tables of *Stenostomum sthenum* and *Macrostomum lignano* with gene name, start and stop positions and codons, length and the direction of transcription (- for the minus strand). (PDF 41 kb)

Additional file 4: tRNA secondary structures in the mitochondrial genome of *Stenostomum sthenum* as predicted by MITOS [20]. (PDF 640 kb)

Additional file 5: tRNA secondary structures in the mitochondrial genome of *Macrostomum lignano* as predicted by MITOS [20]. (PDF 591 kb)

Additional file 6: Hydrophobicity profiles of the six putative atp8 amino acid sequences of free-living flatworms (A-F) and of two representative published atp8 amino acid sequences, from a mollusc (G) and an annelid (H). (TIF 101 kb)

Additional file 7: Analysis of gene order rearrangements using TreeREx [15] and CREx [13], including family diagrams. (PDF 418 kb)

Additional file 8: Mitochondrial gene order in macrostomorphans, showing the newly sequenced complete mitochondrial genome of *Macrostomum lignano* and the partial mitochondrial genome of *Microstomum lineare* [8]. The gene order between these two members of Macrostomorpha is not conserved. (TIF 133 kb)

Additional file 9: Amino acid alignment of atp8 in flatworms and other lophotrochozoans: a gastrotrich (*Lepidodermella squamata*), annelids (*Platynereis dumerilii* and *Sipunculus nudus*), molluscs (*Sepiella japonica* and *Ascobulla fragilis*), a phoronid (*Phoronopsis harmeri*), a kamptozoan (*Loxosomella aloxiata*), a bryozoan (*Bugula neritina*), a brachiopod (*Terebratalia transversa*) and a nemertean (*Micrura bella*). Putative triclad atp8 sequences were extracted from Ross et al. [5]. (TIF 129 kb)

Additional file 10: atp8 alignment matrix without triclads (for details see Fig. 4). (TXT 1 kb)

Additional file 11: atp8 alignment matrix with triclads (for details see Additional file 9). (TXT 4 kb)

## Abbreviations

atp6: ATP synthase F0 subunit 6
atp8: ATP synthase F0 subunit 8
cob: Cytochrome b
cox1: Cytochrome c oxidase subunit I
cox2: Cytochrome c oxidase subunit II
cox3: Cytochrome c oxidase subunit III
nad1: NADH dehydrogenase subunit 1
nad2: NADH dehydrogenase subunit 2
nad3: NADH dehydrogenase subunit 3
nad4: NADH dehydrogenase subunit 4
nad4l: NADH dehydrogenase subunit 4L
nad5: NADH dehydrogenase subunit 5
nad6: NADH dehydrogenase subunit 6
PCR: Polymerase chain reaction
RNAseq: RNA sequencing.
