# Supplementary figures and images for: Atp8 is in the ground pattern of flatworm mitochondrial genomes
Source: BMC Genomics. 2017 May 26;18:414. doi: 10.1186/s12864-017-3807-2 (PMC5446695; doi:10.1186/s12864-017-3807-2)

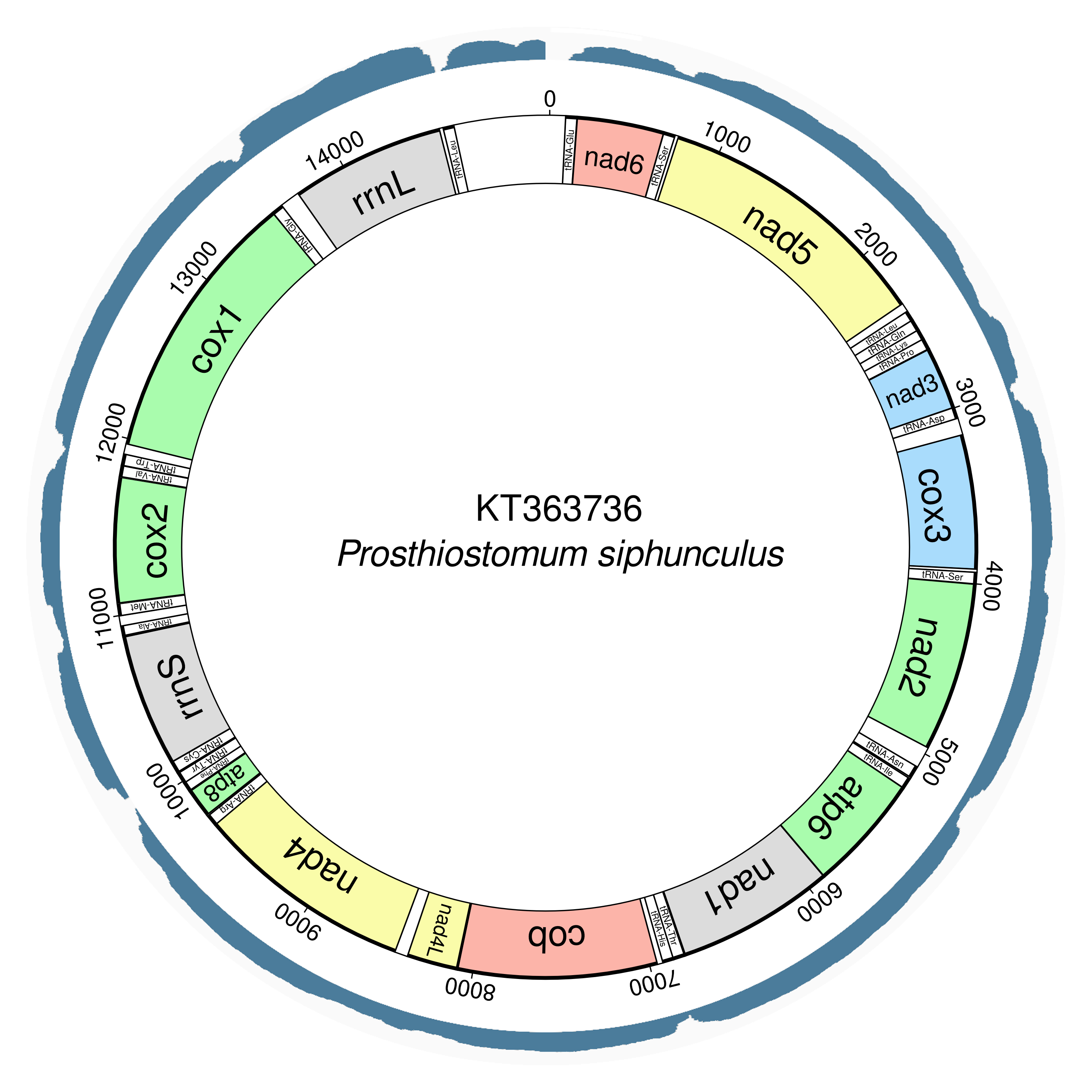

Supplement: Supplementary file 2 — Circular mitochondrial genome of Prosthiostomum siphunculus [2]. Outermost circle displays logarithmically mapped RNAseq reads. tRNA genes are abbreviated with tRNA and three letters and a number if applicable. Colours indicate the widely conserved gene cartridges proposed by Mwinyi et al. [18]. Unlabeled and uncoloured regions are intergenic spacers. (TIF 870 kb) [file 12864_2017_3807_MOESM2_ESM.tif]

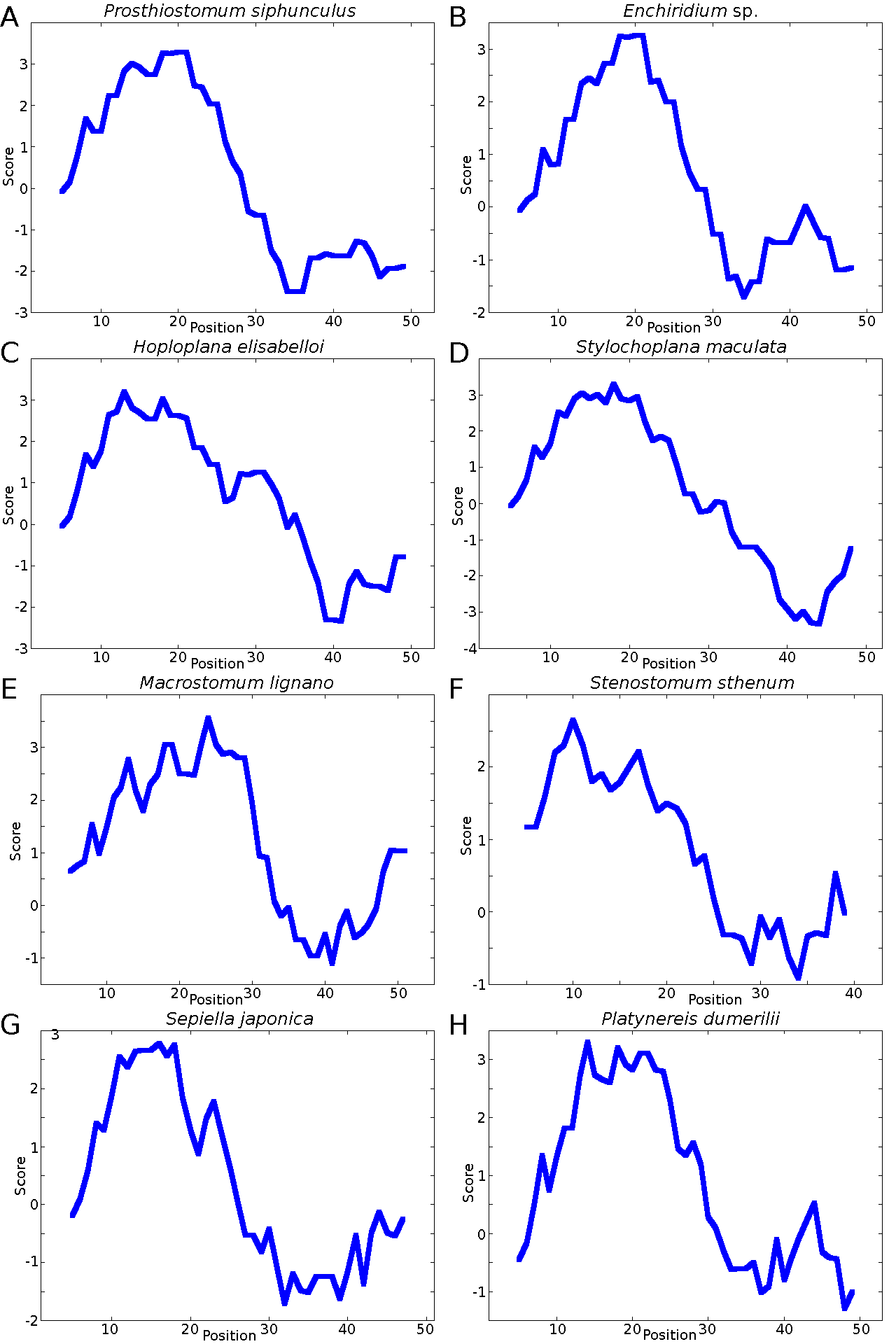

Supplement: Supplementary file 6 — Hydrophobicity profiles of the six putative atp8 amino acid sequences of free-living flatworms (A-F) and of two representative published atp8 amino acid sequences, from a mollusc (G) and an annelid (H). (TIF 101 kb) [file 12864_2017_3807_MOESM6_ESM.tif]

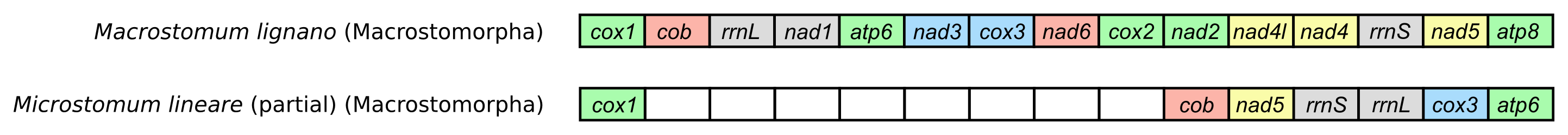

Supplement: Supplementary file 8 — Mitochondrial gene order in macrostomorphans, showing the newly sequenced complete mitochondrial genome of Macrostomum lignano and the partial mitochondrial genome of Microstomum lineare [8]. The gene order between these two members of Macrostomorpha is not conserved. (TIF 133 kb) [file 12864_2017_3807_MOESM8_ESM.tif]

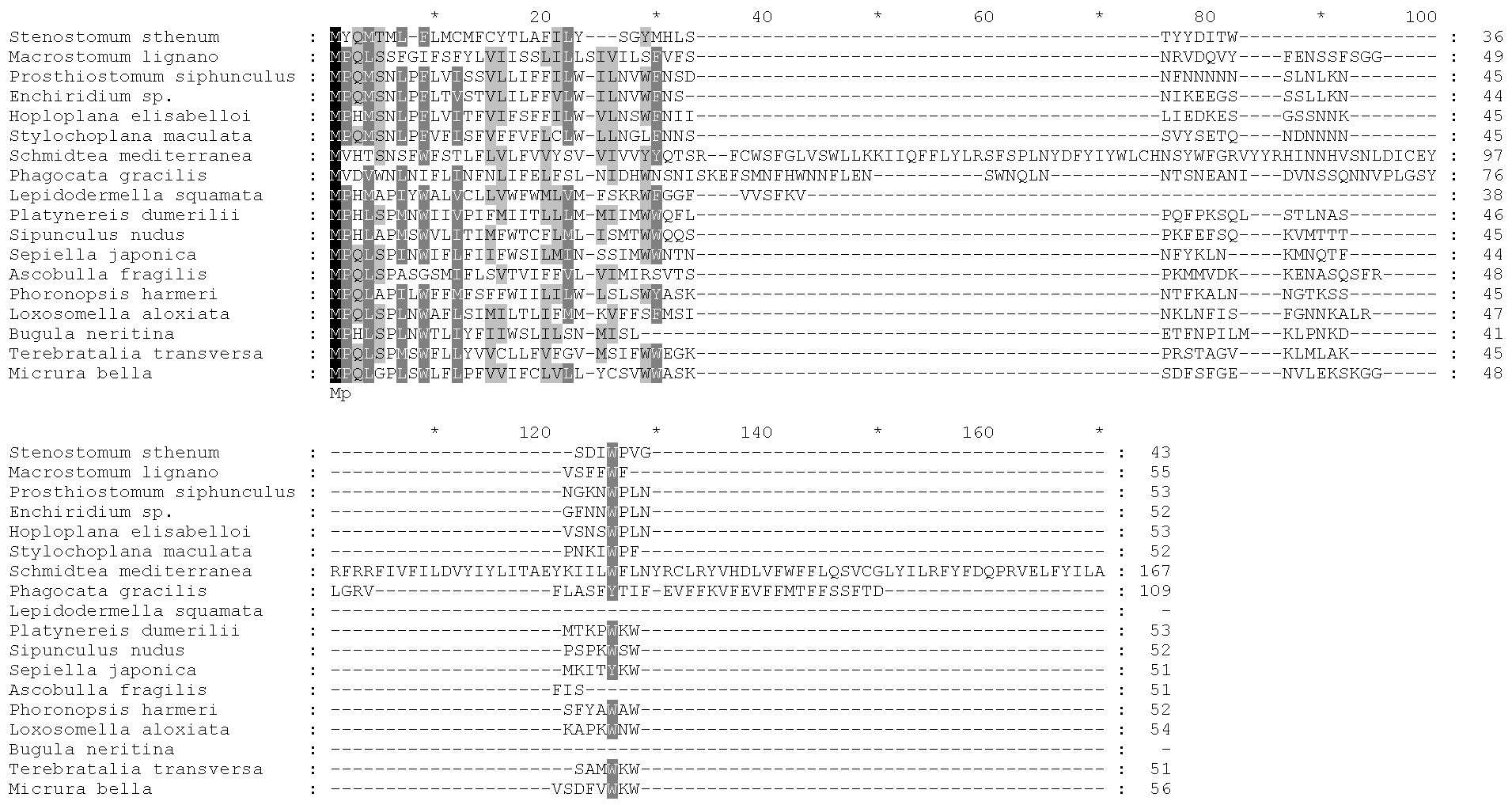

Supplement: Supplementary file 9 — Amino acid alignment of atp8 in flatworms and other lophotrochozoans: a gastrotrich (Lepidodermella squamata), annelids (Platynereis dumerilii and Sipunculus nudus), molluscs (Sepiella japonica and Ascobulla fragilis), a phoronid (Phoronopsis harmeri), a kamptozoan (Loxosomella aloxiata), a bryozoan (Bugula neritina), a brachiopod (Terebratalia transversa) and a nemertean (Micrura bella). Putative triclad atp8 sequences were extracted from Ross et al. [5]. (TIF 129 kb) [file 12864_2017_3807_MOESM9_ESM.tif]
