# Supplementary material for: Atp8 is in the ground pattern of flatworm mitochondrial genomes
Source: BMC Genomics. 2017 May 26;18:414. doi: 10.1186/s12864-017-3807-2 (PMC5446695; doi:10.1186/s12864-017-3807-2)
Supplement: Supplementary file 3 — Gene annotation tables of Stenostomum sthenum and Macrostomum lignano with gene name, start and stop positions and codons, length and the direction of transcription (- for the minus strand). (PDF 41 kb) [file 12864_2017_3807_MOESM3_ESM.pdf]

***Macrostomum lignano***

| Name | Start | Stop | Length | Start codon | Stop codon | Strand |
|---|---|---|---|---|---|---|
| cox1 | 1 | 1548 | 1548 | att | taa | + |
| intergenic spacer | 1549 | 1559 | 11 | | | |
| cob | 1560 | 2660 | 1101 | att | tag | + |
| trnH(gtg) | 2661 | 2720 | 60 | | | + |
| trnQ(ttg) | 2721 | 2775 | 55 | | | + |
| trnW(tca) | 2776 | 2837 | 62 | | | + |
| trnT(tgt) | 2838 | 2896 | 59 | | | + |
| trnP(tgg) | 2897 | 2959 | 63 | | | + |
| intergenic spacer | 2960 | 2968 | 9 | | | |
| rrnL | 2969 | 3835 | 867 | | | + |
| intergenic spacer | 3836 | 3845 | 10 | | | |
| trnV(tac) | 3846 | 3905 | 60 | | | + |
| trnE(ttc) | 3906 | 3965 | 60 | | | + |
| trnS1(gct) | 3963 | 4015 | 53 | | | + |
| nad1 | 4015 | 4887 | 873 | ttg | taa | + |
| intergenic spacer | 4888 | 4912 | 25 | | | |
| atp6 | 4913 | 5590 | 678 | ttg | taa | + |
| intergenic spacer | 5591 | 5600 | 10 | | | |
| nad3 | 5601 | 5953 | 353 | att | ta | + |
| intergenic spacer | 5954 | 5985 | 32 | | | |
| cox3 | 5986 | 6774 | 789 | gtt | taa | + |
| intergenic spacer | 6775 | 6776 | 2 | | | |
| nad6 | 6777 | 7214 | 438 | atg | taa | + |
| trnM(cat) | 7213 | 7272 | 60 | | | + |
| trnA(tgc) | 7273 | 7334 | 62 | | | + |
| trnG(tcc) | 7335 | 7395 | 61 | | | + |
| cox2 | 7396 | 8070 | 675 | ata | tag | + |
| trnK(ctt) | 8069 | 8121 | 53 | | | + |
| trnF(gaa) | 8122 | 8183 | 62 | | | + |
| nad2 | 8181 | 9071 | 891 | gtg | tag | + |
| trnI(gat) | 9072 | 9133 | 62 | | | + |
| nad4l | 9133 | 9390 | 258 | atg | taa | + |
| trnS2(tga) | 9390 | 9443 | 54 | | | + |
| intergenic spacer | 9444 | 10165 | 722 | | | |
| nad4 | 10166 | 11392 | 1227 | ttg | taa | + |
| trnD(gtc) | 11391 | 11448 | 58 | | | + |
| trnR(tcg) | 11449 | 11508 | 60 | | | + |
| rrnS | 11450 | 12188 | 739 | | | + |
| intergenic spacer | 12189 | 12204 | 16 | | | |
| nad5 | 12205 | 13761 | 1557 | atg | taa | + |
| trnN(gtt) | 13760 | 13819 | 60 | | | + |
| trnY(gta) | 13819 | 13880 | 62 | | | + |
| trnL2(taa) | 13881 | 13940 | 60 | | | + |
| trnC(gca) | 13941 | 13999 | 59 | | | + |
| intergenic spacer | 14000 | 14012 | 13 | | | |
| atp8 | 14013 | 14180 | 168 | ata | taa | + |
| intergenic spacer | 14181 | 14193 | 13 | | | |

***Stenostomum sthenum***

| Name | Start | Stop | Length | Start codon | Stop codon | Strand |
|---|---|---|---|---|---|---|
| cox1 | 1 | 1557 | 1557 | atc | tag | + |
| intergenic spacer | 1558 | 1566 | 9 | | | |
| trnA(tgc) | 1567 | 1623 | 57 | | | + |
| trnK(ttt) | 1624 | 1687 | 64 | | | + |
| intergenic spacer | 1688 | 1695 | 8 | | | |
| trnE(ttc) | 1696 | 1759 | 64 | | | + |
| intergenic spacer | 1760 | 1760 | 1 | | | |
| trnL2(taa) | 1761 | 1825 | 65 | | | + |
| intergenic spacer | 1826 | 1828 | 3 | | | |
| trnH(gtg) | 1829 | 1889 | 61 | | | - |
| intergenic spacer | 1890 | 1939 | 50 | | | |
| rrnS | 1940 | 2618 | 679 | | | - |
| intergenic spacer | 2619 | 2638 | 20 | | | |
| trnV(tac) | 2639 | 2698 | 60 | | | - |
| nad3-1 | 2699 | 3062 | 364 | atg | t | - |
| intergenic spacer | 3063 | 3114 | 52 | | | |
| trnL1(tag) | 3115 | 3174 | 60 | | | - |
| intergenic spacer | 3175 | 3179 | 5 | | | |
| trnM(cat) | 3180 | 3245 | 66 | | | - |
| intergenic spacer | 3246 | 3247 | 2 | | | |
| trnI(gat) | 3248 | 3306 | 59 | | | + |
| intergenic spacer | 3307 | 4146 | 840 | | | |
| atp8 | 4147 | 4276 | 130 | atc | t | - |
| intergenic spacer | 4277 | 4424 | 148 | | | |
| trnN(gtt) | 4425 | 4484 | 60 | | | + |
| cob | 4485 | 5579 | 1095 | atc | tag | + |
| intergenic spacer | 5580 | 5585 | 6 | | | |
| trnP(tgg) | 5586 | 5649 | 64 | | | + |
| nad6 | 5650 | 6153 | 504 | att | tag | + |
| trnS1(gct) | 6119 | 6173 | 55 | | | + |
| cox3 | 6174 | 6953 | 780 | atc | taa | + |
| intergenic spacer | 6954 | 6963 | 10 | | | |
| nad1 | 6964 | 7860 | 897 | atg | taa | + |
| intergenic spacer | 7861 | 7862 | 2 | | | |
| trnY(gta) | 7863 | 7924 | 62 | | | + |
| intergenic spacer | 7925 | 7926 | 2 | | | |
| trnC(gca) | 7927 | 7989 | 63 | | | + |
| intergenic spacer | 7990 | 8015 | 26 | | | |
| rrnL | 8016 | 8965 | 950 | | | + |
| intergenic spacer | 8966 | 9030 | 65 | | | |
| trnI(gat) | 9031 | 9089 | 59 | | | - |
| intergenic spacer | 9090 | 9091 | 2 | | | |
| trnM(cat) | 9092 | 9157 | 66 | | | + |
| intergenic spacer | 9158 | 9162 | 5 | | | |
| trnL1(tag) | 9163 | 9222 | 60 | | | + |
| intergenic spacer | 9223 | 9274 | 52 | | | |
| nad3-0 | 9275 | 9638 | 364 | atg | t | + |
| trnV(tac) | 9639 | 9698 | 60 | | | + |
| intergenic spacer | 9699 | 9718 | 20 | | | |
| rrnS | 9719 | 10397 | 679 | | | + |

| | | | | | | |
|---|---|---|---|---|---|---|
| intergenic spacer | 10398 | 10447 | 50 | | | |
| trnH(gtg) | 10448 | 10508 | 61 | | | + |
| intergenic spacer | 10509 | 10511 | 3 | | | |
| trnL2(taa) | 10512 | 10576 | 65 | | | - |
| intergenic spacer | 10577 | 10577 | 1 | | | |
| trnE(ttc) | 10578 | 10641 | 64 | | | - |
| intergenic spacer | 10642 | 10649 | 8 | | | |
| trnK(ttt) | 10650 | 10713 | 64 | | | - |
| trnA(tgc) | 10714 | 10770 | 57 | | | - |
| intergenic spacer | 10771 | 10801 | 31 | | | |
| cox2 | 10802 | 11479 | 678 | ata | taa | - |
| intergenic spacer | 11480 | 11540 | 61 | | | |
| trnM(cat) | 11541 | 11606 | 66 | | | - |
| nad4l | 11602 | 11859 | 258 | ata | taa | - |
| intergenic spacer | 11860 | 11866 | 7 | | | |
| trnW(tca) | 11867 | 11930 | 64 | | | + |
| intergenic spacer | 11931 | 11931 | 1 | | | |
| trnQ(ttg) | 11932 | 11994 | 63 | | | - |
| intergenic spacer | 11995 | 11996 | 2 | | | |
| trnT(tgt) | 11997 | 12059 | 63 | | | - |
| intergenic spacer | 12060 | 12061 | 2 | | | |
| nad4 | 12062 | 13294 | 1233 | ata | taa | + |
| trnS2(tga) | 13293 | 13354 | 62 | | | - |
| intergenic spacer | 13355 | 13357 | 3 | | | |
| trnD(gtc) | 13358 | 13420 | 63 | | | + |
| atp6 | 13421 | 14086 | 666 | ata | taa | + |
| intergenic spacer | 14087 | 14089 | 3 | | | |
| nad5 | 14090 | 15721 | 1632 | att | tag | + |
| trnF(gaa) | 15696 | 15755 | 60 | | | + |
| intergenic spacer | 15756 | 15787 | 32 | | | |
| nad2 | 15788 | 16750 | 963 | att | taa | + |
| trnG(tcc) | 16749 | 16809 | 61 | | | + |
| trnR(tcg) | 16808 | 16866 | 59 | | | - |
| intergenic spacer | 16867 | 16944 | 78 | | | |
