# Supplementary material for: Atp8 is in the ground pattern of flatworm mitochondrial genomes
Source: BMC Genomics. 2017 May 26;18:414. doi: 10.1186/s12864-017-3807-2 (PMC5446695; doi:10.1186/s12864-017-3807-2)
Supplement: Supplementary file 7 — Analysis of gene order rearrangements using TreeREx [15] and CREx [13], including family diagrams. (PDF 418 kb) [file 12864_2017_3807_MOESM7_ESM.pdf]

TreeREx analysis, gene order for node A7 is the provided putative lophotrochozoan ground pattern:

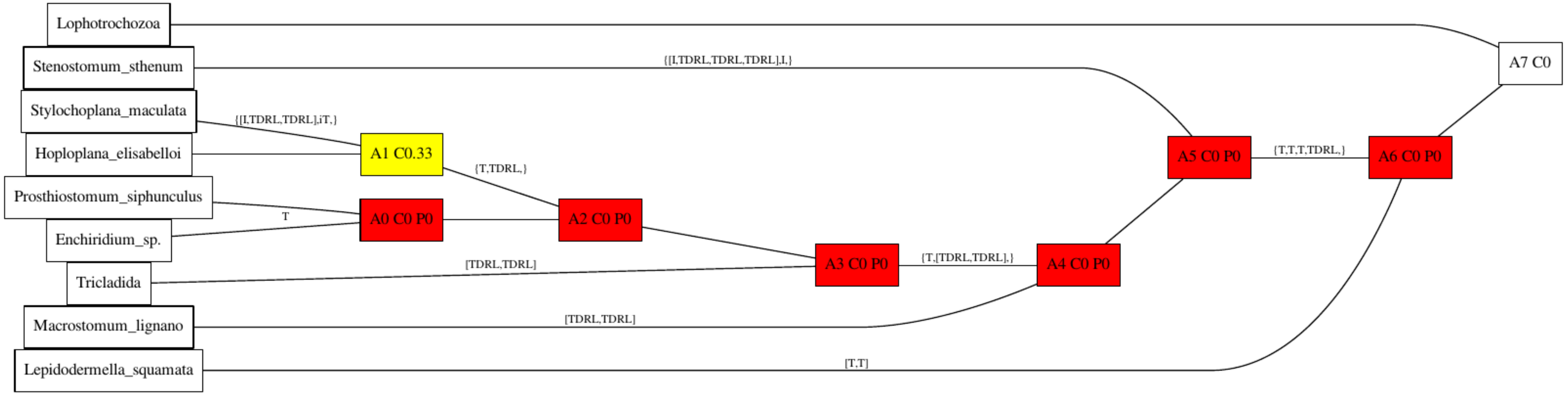

>A7
cox1 cox2 atp8 atp6 rrnS rrnL nad1 nad6 cob nad4l nad4 nad5 cox3 nad3 nad2
>A6
cox1 nad1 cob nad4l nad4 atp8 atp6 rrnS rrnL nad6 nad5 cox2 cox3 nad3 nad2
>A5
cox1 nad3 cox3 nad2 cob nad4l nad4 rrnS rrnL nad6 nad5 nad1 atp6 atp8 cox2
>A4
cox1 nad3 cox3 nad2 cob nad4l nad4 rrnS rrnL nad6 nad5 nad1 atp6 atp8 cox2
>A3
cox1 rrnL nad6 nad5 nad3 cox3 nad2 atp6 nad1 cob nad4l nad4 atp8 rrnS cox2
>Tricladida
cox1 nad6 nad5 cox3 atp6 nad1 cox2 nad3 nad2 atp8 rrnS rrnL cob nad4l nad4
>A2
cox1 rrnL nad6 nad5 nad3 cox3 nad2 atp6 nad1 cob nad4l nad4 atp8 rrnS cox2
>A0
cox1 rrnL nad6 nad5 nad3 cox3 nad2 atp6 nad1 cob nad4l nad4 atp8 rrnS cox2
>Prosthiostomum_siphunculus
cox1 rrnL nad6 nad5 nad3 cox3 nad2 atp6 nad1 cob nad4l nad4 atp8 rrnS cox2

>Enchiridium_sp.
cox1 rrnL nad6 nad5 nad3 cox3 nad2 atp6 nad1 cob atp8 rrnS nad4l nad4 cox2
>A1
cox1 rrnL nad6 nad5 cox3 nad3 nad2 atp6 cob nad4 nad1 nad4l atp8 rrnS cox2
>Hoploplana_elisabelloi
cox1 rrnL nad6 nad5 cox3 nad3 nad2 atp6 cob nad4 nad1 nad4l atp8 rrnS cox2
>Stylochoplana_maculata
cox1 cox3 atp8 nad1 nad2 nad4l rrnS nad6 nad5 nad3 atp6 cob nad4 rrnL cox2
>Macrostomum_lignano
cox1 cob rrnL nad1 atp6 nad3 cox3 nad6 cox2 nad2 nad4l nad4 rrnS nad5 atp8
>Stenostomum_sthenum
cox1 -atp8 cob nad6 cox3 nad1 rrnL nad3 rrnS -cox2 -nad4l nad4 atp6 nad5 nad2
>Lepidodermella_squamata
cox1 nad1 nad6 cob nad4l nad4 nad5 atp8 atp6 rrnS rrnL cox2 cox3 nad3 nad2
>Lophotrochozoa
cox1 cox2 atp8 atp6 rrnS rrnL nad1 nad6 cob nad4l nad4 nad5 cox3 nad3 nad2
scenario to A7
Empty

scenario to A6
Empty

scenario to A5
unordered{
transposition(cox2 atp8 atp6 rrnS rrnL nad1 nad6 cob nad4l nad4 nad5 ,cox3 nad3 nad2 ,)
, transposition(atp8 ,atp6 ,)
, transposition(cox3 ,nad3 ,)
, TDRL(rrnS rrnL nad6 cob nad4l nad4 nad5 ,atp8 atp6 nad1 ,)
,} complete=1

scenario to A4
Empty

scenario to A3

```
unordered{
	transposition(atp6 ,nad1 ,)
,	ordered[
		TDRL(atp8 rrnL nad6 cob nad4l nad4 nad5 ,atp6 rrnS nad1 cox3 nad3 nad2 ,)
,		TDRL(atp6 rrnL nad1 nad6 nad5 cox3 nad3 nad2 ,atp8 rrnS cob nad4l nad4 ,)
	] complete=1
,} complete=1

scenario to Tricladida
ordered[
	TDRL(cox2 atp6 nad1 cob nad4l nad4 cox3 ,atp8 rrnS nad3 nad2 ,)
,	TDRL(cox2 atp8 atp6 rrnS nad1 nad6 nad5 cox3 nad3 nad2 ,rrnL cob nad4l nad4 ,)
] complete=1

scenario to A2
Empty

scenario to A0
Empty

scenario to Prosthiostomum_siphunculus
Empty

scenario to Enchiridium_sp.
transposition(atp8 rrnS ,nad4l nad4 ,)

scenario to A1
unordered{
	transposition(cox3 ,nad3 ,)
,	TDRL(cob nad4 ,nad1 nad4l ,)
,} complete=1

scenario to Hoploplana_elisabelloi
Empty
```

```
scenario to Stylochoplana_maculata
unordered{
        ordered[
                inversion(atp8 atp6 rrnS nad1 nad6 cob nad4l nad4 nad5 cox3 nad3 nad2 )
,               TDRL(atp6 nad1 nad6 cob nad4 nad5 nad3 ,atp8 rrnS nad4l cox3 nad2 ,)
,               TDRL(atp6 rrnS nad6 cob nad4l nad4 nad5 nad3 nad2 ,atp8 nad1 ,)
        ] complete=1
,       inverse transposition(atp8 atp6 rrnS nad1 nad6 cob nad4l nad4 nad5 cox3 nad3 nad2 ,rrnL ,)
,} complete=1

scenario to Macrostomum_lignano
ordered[
        TDRL(atp8 atp6 rrnS rrnL nad1 cob nad4l nad4 nad5 nad2 ,nad6 cox3 nad3 ,)
,       TDRL(cox2 atp6 rrnL nad1 nad6 cob cox3 nad3 ,atp8 rrnS nad4l nad4 nad5 nad2 ,)
] complete=1

scenario to Stenostomum_sthenum
unordered{
        ordered[
                inversion(cox2 atp8 )
,               TDRL(cox2 atp8 rrnS nad1 nad5 ,atp6 rrnL nad6 cob nad4l nad4 nad2 ,)
,               TDRL(cox2 atp8 atp6 rrnS nad6 cob nad4l nad4 ,rrnL nad1 nad5 cox3 nad2 ,)
,               TDRL(atp8 rrnL nad1 nad6 cob cox3 ,cox2 atp6 rrnS nad4l nad4 nad5 nad3 nad2 ,)
        ] complete=1
,       inversion(nad4l )
,} complete=1

scenario to Lepidodermella_squamata
ordered[
        transposition(atp8 atp6 rrnS rrnL nad6 ,cob nad4l nad4 ,)
,       transposition(atp8 atp6 rrnS rrnL ,nad6 cob nad4l nad4 nad5 ,)
] complete=1
```

scenario to Lophotrochozoa
Empty

CREx comparisons:

Lophotrochozoa → Lepidodermella_squamata

- family diagram for Lophotrochozoa **(e)**

cox1	cox2	atp8	atp6	rrnS	rrnL	nad1	nad6	cob	nad4l	nad4	nad5	cox3	nad3	nad2

- family diagram for Lepidodermella_squamata **(e)**

cox1	nad1	nad6	cob	nad4l	nad4	nad5	atp8	atp6	rrnS	rrnL	cox2	cox3	nad3	nad2

- scenario:
  - reversal

cox2	**(e)**
-cox2	**(e)**

  - reversal

atp8	atp6	rrnS	rrnL	**(e)**
-rrnL	-rrnS	-atp6	-atp8	**(e)**

  - reversal

nad1	nad6	cob	nad4l	nad4	nad5	**(e)**
-nad5	-nad4	-nad4l	-cob	-nad6	-nad1	**(e)**

  - reversal

-cox2	-rrnL	-rrnS	-atp6	-atp8	-nad5	-nad4	-nad4l	-cob	-nad6	-nad1	**(e)**
nad1	nad6	cob	nad4l	nad4	nad5	atp8	atp6	rrnS	rrnL	cox2	**(e)**

Lophotrochozoa → Stenostomum_sthenum

- family diagram for Lophotrochozoa **(e)**

cox1	cox2	atp8	atp6	rrnS	rrnL	nad1	nad6	cob	nad4l	nad4	nad5	cox3	nad3	nad2

- family diagram for Stenostomum_sthenum **(e)**

cox1	-atp8	cob	nad6	cox3	nad1	rrnL	nad3	rrnS	-cox2	-nad4l	nad4	atp6	nad5	nad2

- scenario:
  - transposition

rrnL nad1	**(e)**
nad1 rrnL	**(e)**

  - transposition

nad6 cob	**(e)**
cob nad6	**(e)**

  - reversal

nad4l	**(e)**
-nad4l	**(e)**

  -

reversal	cox2 atp8 atp6 rrnS nad1 rrnL cob nad6 -nad4l nad4 nad5 cox3 nad3	**(e)**
→	-atp8 -cox2 atp6 rrnS nad1 rrnL cob nad6 -nad4l nad4 nad5 cox3 nad3	**(e)**
tdrl	-atp8 -cox2 atp6 rrnS nad1 rrnL cob nad6 -nad4l nad4 nad5 cox3 nad3	**(e)**
→	-atp8 atp6 rrnS cob nad6 nad5 cox3 -cox2 nad1 rrnL -nad4l nad4 nad3	**(e)**
tdrl	-atp8 atp6 rrnS cob nad6 nad5 cox3 -cox2 nad1 rrnL -nad4l nad4 nad3	**(e)**
→	-atp8 atp6 cob nad6 nad5 cox3 nad1 rrnL nad3 rrnS -cox2 -nad4l nad4	**(e)**
tdrl	-atp8 atp6 cob nad6 nad5 cox3 nad1 rrnL nad3 rrnS -cox2 -nad4l nad4	**(e)**
→	-atp8 cob nad6 cox3 nad1 rrnL nad3 rrnS -cox2 -nad4l nad4 atp6 nad5	**(e)**

Lophotrochozoa → Macrostomum_lignano

- family diagram for Lophotrochozoa **(e)**

cox1	cox2	atp8	atp6	rrnS	rrnL	nad1	nad6	cob	nad4l	nad4	nad5	cox3	nad3	nad2	

- family diagram for Macrostomum_lignano **(e)**

cox1	cob	rrnL	nad1	atp6	nad3	cox3	nad6	cox2	nad2	nad4l	nad4	rrnS	nad5	atp8

- scenario:
  - transposition

cox3	nad3	**(e)**
nad3	cox3	**(e)**

  -

tdrl	cox2	atp8	atp6	rrnS	rrnL	nad1	nad6	cob	nad4l	nad4	nad5	nad3	cox3	nad2	**(e)**
→	cox2	atp6	rrnS	cob	nad5	nad3	cox3	nad2	atp8	rrnL	nad1	nad6	nad4l	nad4	**(e)**
tdrl	cox2	atp6	rrnS	cob	nad5	nad3	cox3	nad2	atp8	rrnL	nad1	nad6	nad4l	nad4	**(e)**
→	cox2	cob	nad2	rrnL	nad1	nad4l	nad4	atp6	rrnS	nad5	nad3	cox3	atp8	nad6	**(e)**
tdrl	cox2	cob	nad2	rrnL	nad1	nad4l	nad4	atp6	rrnS	nad5	nad3	cox3	atp8	nad6	**(e)**
→	cob	rrnL	nad1	atp6	nad3	cox3	nad6	cox2	nad2	nad4l	nad4	rrnS	nad5	atp8	**(e)**

Lophotrochozoa → Tricladida

- family diagram for Lophotrochozoa **(e)**

  cox1 cox2 atp8 atp6 rrnS rrnL nad1 nad6 cob nad4l nad4 nad5 cox3 nad3 nad2

- family diagram for Tricladida **(e)**

  cox1 nad6 nad5 cox3 atp6 nad1 cox2 nad3 nad2 atp8 rrnS rrnL cob nad4l nad4

- scenario:

tdrl	cox2 atp8 atp6 rrnS rrnL nad1 nad6 cob nad4l nad4 nad5 cox3 nad3 nad2	**(e)**
→	cox2 nad6 nad5 cox3 nad3 nad2 atp8 atp6 rrnS rrnL nad1 cob nad4l nad4	**(e)**
tdrl	cox2 nad6 nad5 cox3 nad3 nad2 atp8 atp6 rrnS rrnL nad1 cob nad4l nad4	**(e)**
→	nad6 nad5 cox3 atp6 nad1 cox2 nad3 nad2 atp8 rrnS rrnL cob nad4l nad4	**(e)**
