# Supplementary material for: Atp8 is in the ground pattern of flatworm mitochondrial genomes
Source: BMC Genomics. 2017 May 26;18:414. doi: 10.1186/s12864-017-3807-2 (PMC5446695; doi:10.1186/s12864-017-3807-2)
Supplement: Supplementary file 4 — tRNA secondary structures in the mitochondrial genome of Stenostomum sthenum as predicted by MITOS [20]. (PDF 640 kb) [file 12864_2017_3807_MOESM4_ESM.pdf]

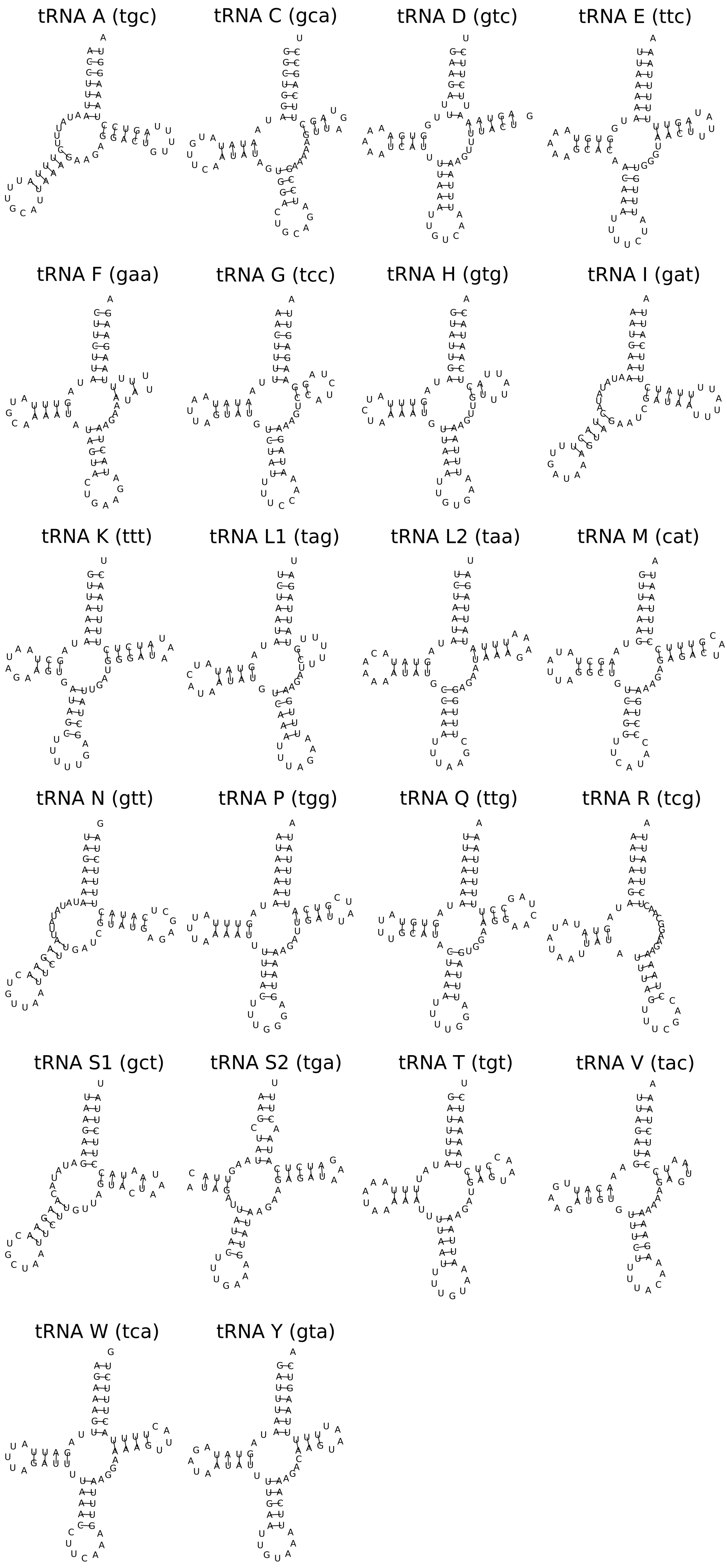
tRNA A (tgc)
tRNA C (gca)
tRNA D (gtc)
tRNA E (ttc)
tRNA F (gaa)
tRNA G (tcc)
tRNA H (gtg)
tRNA I (gat)
tRNA K (ttt)
tRNA L1 (tag)
tRNA L2 (taa)
tRNA M (cat)
tRNA N (gtt)
tRNA P (tgg)
tRNA Q (ttg)
tRNA R (tcg)
tRNA S1 (gct)
tRNA S2 (tga)
tRNA T (tgt)
tRNA V (tac)
tRNA W (tca)
tRNA Y (gta)
